# Isolinderalactone regulates macrophage polarization and efferocytosis by activating the LXRα pathway against ulcerative colitis

**DOI:** 10.1186/s13020-025-01216-9

**Published:** 2025-10-01

**Authors:** Mincong Huang, Mengyao Lan, Xin Liu, Cailu Lin, Lulu Zeng, Ying Li, Feng Li, Xiaotong Dou, Yan Zhao, Yuan Shi, Xiangwei Xu, Jinfeng Sun, Guang Liang

**Affiliations:** 1 Affiliated Yongkang First People’s Hospital and School of Pharmacy, Hangzhou Medical College, Hangzhou, 310014 Zhejiang China; 2 Zhejiang TCM Key Laboratory of Pharmacology and Translational Research of Natural Products, School of Pharmacy, Hangzhou Medical College, Hangzhou, 311399 Zhejiang China; 3 Department of Colorectal Surgery, Zhejiang Provincial People’s Hospital, Affiliated People’s Hospital of Hangzhou Medical College, Hangzhou, 310014 Zhejiang People’s Republic of China; 4 Key Laboratory of Natural Medicines of the Changbai Mountain, Ministry of Education, College of Pharmacy, Yanbian University, Yanji , 133002 Jilin China

**Keywords:** *Lindera aggregate*, Isolinderalactone, Liver X receptor α, Macrophage polarization, Efferocytosis, Ulcerative colitis

## Abstract

**Background:**

Ulcerative colitis (UC), a chronic inflammatory bowel disease, remains an unmet medical need. *Lindera aggregata,* a traditional Chinese medicine used in treating gastrointestinal disorders, has demonstrated anti-UC efficacy, though its bioactive components are poorly characterized. Isolinderalactone (ILDL), a characteristic sesquiterpene lactone isolated from *Lindera aggregata*, has been demonstrated anti-cancer properties. However, its therapeutic potential in UC remains unexplored.

**Methods:**

Lipopolysaccharide (LPS)-induced RAW264.7 inflammatory cell model was used to screen the anti-inflammatory properties of *Lindera aggregata*'s characteristic compounds in vitro. DSS induced UC mouse model was used to study the anti-UC efficacy of ILDL in vivo. Transcriptomic was used to explore the anti-inflammatory mechanism of ILDL. Drug affinity responsible target stability was used to identify the combination of the ILDL and LXRα. LXR-mediated effects were further assessed via flow cytometry and Western blotting.

**Results:**

ILDL effectively inhibits macrophage polarization and the production of inflammatory mediators in vitro, and improves symptoms and tissue lesions in acute UC mice in *vivo*. Transcriptomic analysis revealed the involvement of the LXR-mediated pathway in ILDL’s effects. Furthermore, ILDL was able to bind to LXRα and to upregulate LXRα target genes expression such as ABCA1, suggesting that ILDL itself can activate the LXRα pathway. Genetic/pharmacological LXRα inhibition abrogated ILDL's anti-inflammatory effects, confirming an LXRα-dependent mechanism. In addition to inhibiting macrophage M1 polarization, the activation of LXRα by ILDL can also promote macrophage efferocytosis of apoptotic intestinal epithelial cells in the co-culture system.

**Conclusions:**

ILDL activates the LXRα pathway, inhibiting macrophage M1 polarization, reducing pro-inflammatory mediators production, and promoting macrophage efferocytosis. ILDL is a promising candidate compound from *Lindera aggregata* for anti-inflammation and UC treatment.

**Graphical Abstract:**

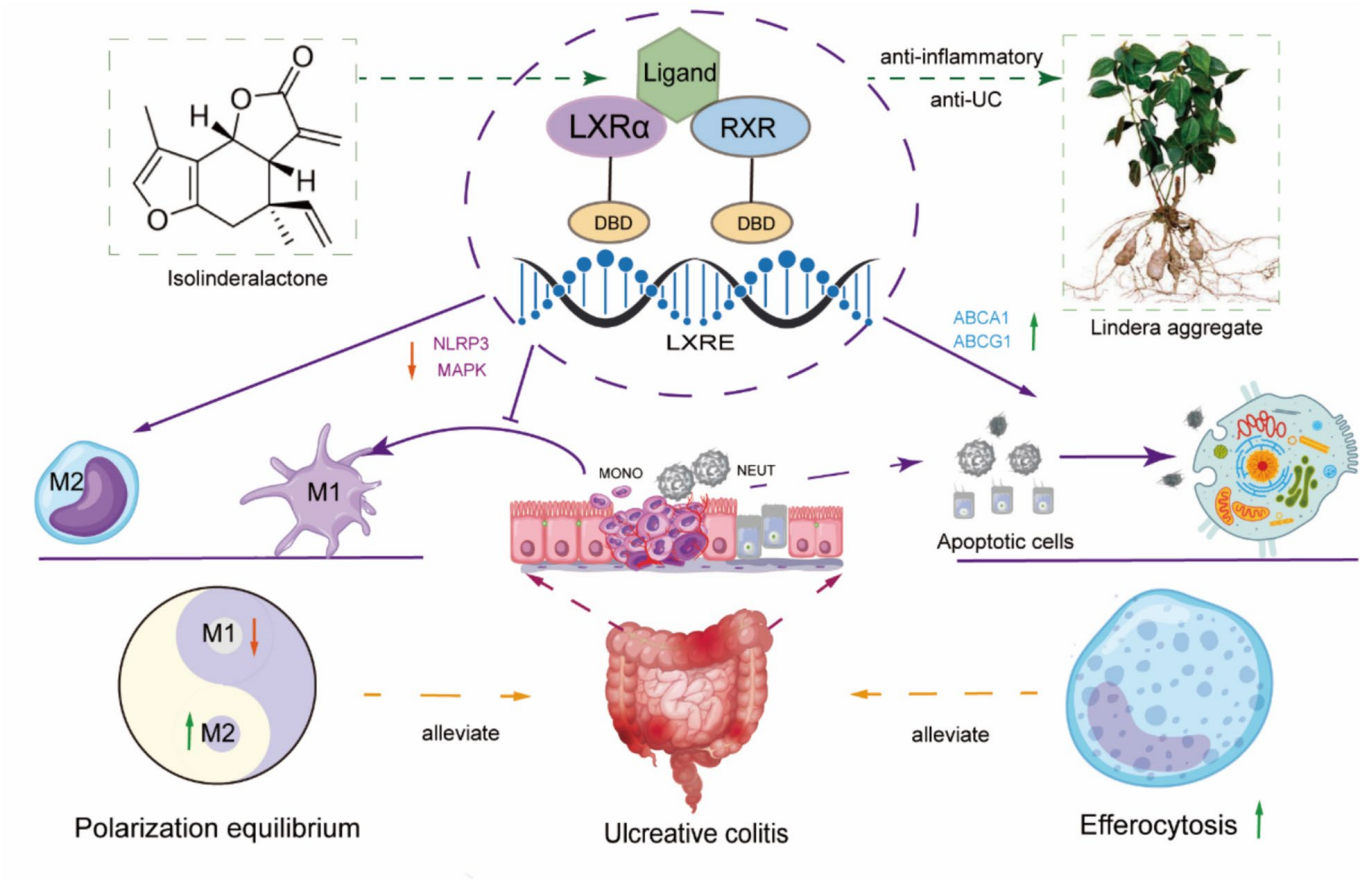

**Supplementary Information:**

The online version contains supplementary material available at 10.1186/s13020-025-01216-9.

## Introduction

Ulcerative colitis (UC) is a chronic non-specific inflammatory disease primarily affecting the submucosa and mucosa of the rectum and colon. Epidemiologic studies indicate a global prevalence of approximately 5 million cases in 2023, with a continuing rise attributed to changes in social environments, lifestyles, and dietary patterns [1]. UC poses a significant challenge to global health due to its detrimental impact on patient quality of life and potentially life-threatening complications [2, 3]. Therefore, it has become a global health challenge over the past two decades [4, 5]. However, current treatment modalities and medications fall short of meeting clinical demands, necessitating ongoing research and development efforts. The main pathological mechanisms underlying UC include impairment of the intestinal epithelial barrier, intestinal flora disturbance, and disruption of immune balance, with immune imbalance being a significant contributor [6, 7].

Macrophages, as crucial innate immune cells, exhibit remarkable plasticity and multifunctionality, playing a central role in immune regulation and tissue repair [8]. Modern medicine implicates macrophage polarization imbalance and impaired efferocytosis in UC pathogenesis. External factors, such as dietary factors, can damage the intestinal barrier, leading to increased intestinal permeability. Metabolites such as enteric microorganisms and LPS invade the intestinal mucosa lamina propria, prompting polarization towards the M1 pro-inflammatory phenotype. M1 macrophages secret large amounts of inflammatory cytokines and chemokines, such as TNF-α and IL-8, which recruit large numbers of neutrophils [9]. While initially crucial for pathogen clearance, excessive inflammation can precipitate widespread apoptosis of intestinal epithelial cells and neutrophils [10]. Macrophages then play a pivotal role in phagocytosing apoptotic cells, activating efferocytosis to prevent secondary necrosis, avoid tissue inflammation, and promote tissue repair [11–13]. Dysregulated macrophage polarization and defective efferocytosis contribute to the occurrence and progression of UC [14]. This highlights the importance of strategies aimed at modulating macrophage polarization balance and maintaining efferocytosis for effective UC management [15–17].

*Lindera aggregata*, a member of the Lauraceae family, is widely distributed in China (Wu Yao) and Japan (Uyaku). According to “Compendium of Materia Medica”, *Lindera aggregata* is extensively used in traditional Chinese medicine as an anodyne and antispasmodic for treating symptoms such as abdominal distension, pain, and indigestion [18]. Our previous research demonstrated that the ethanol extract of *Lindera aggregata* significantly inhibits the production of inflammatory mediators by macrophages, exhibiting good anti-inflammatory effects in vitro. Additionally, it noticeably ameliorates symptoms and histopathological changes in models of UC induced by dextran sodium sulfate (DSS) and 2,4,6-trinitrobenzene sulfonic acid (TNBS), showcasing promising anti-UC effects [19]. However, the specifically anti-inflammatory and anti-UC active compounds of *Lindera aggregata* remain unidentified and warrant further exploration. Therefore, we investigated the anti-inflammatory activity of *Lindera aggregata’s* characteristic compounds using the LPS-induced RAW264.7 inflammatory cell model, identifying isolinderalactone (ILDL) as the most potent anti-inflammatory agent among numerous known components of *Lindera aggregata*. ILDL, the primary sesquiterpene lactone isolated from *Lindera aggregata*, has been previously studied for its anti-cancer properties [20, 21]. However, its anti-inflammatory mechanism and efficacy in UC treatment remain unclear. Thus, in this study, we investigated the anti-inflammatory mechanism of ILDL and its pharmacological effects on UC. Overall, this study aimed to clarify ILDL's anti-inflammatory and anti-UC effects, uncover its mechanism of action, identify and enhance the pharmacological profile of *Lindera aggregata*, and provide essential pharmacological insights to support its future development and utilization.

## Materials and methods

### Drugs and reagents

A list of reagents used in this study is provided in Supplementary Table S1. Isolinderalactone (ILDL) dissolved in DMSO as a high concentration reserve solution for cell experimental studies.

### Cell culture and viability

RAW264.7 cells and MODE-K cells were cultured at 37 °C in a humidified 5% CO_2_ incubator using Dulbecco's Modified Eagle's Medium supplemented with 10% FBS. We seeded RAW264.7 cells in 96-well microplates at a density of 1 × 10^5^ cells/mL, and tested different concentrations of monomeric compounds on the cells. Cell Counting Kit 8 assay (CCK8) was added to each well and incubated for an additional 4 h. Microplate readers were used to quantify 450 nm light absorbance. The cell apoptosis and survival rate were further verified by Annexin V-APC/PI assay.

### Macrophage polarization and Inflammatory medium detection

RAW264.7 cells were seeded on 12-well culture plates at a density of 5 × 10^5^ cells/mL. Then the cells were stimulated with 200 ng/mL LPS and various concentrations of ILDL and incubated for 48 h total. Inflammatory mediators in cells culture supernatants were analyzed by using Cytometric Bead Array (BD Bioscience, CA, USA) and the Total Nitric Oxide Assay Kit (Beyotime Bio-tech, Shanghai, China). In the meantime, RAW264.7 cells were collected, washed, and incubated with anti-CD16/32 antibody for 15 min, then anti-CD86 antibody for 30 min away from light at room temperature. Finally, the cells are washed and detected by flow cytometry.

### Cell co-culture in the transwell co-culture system

MODE-K cells were seeded on the lower chamber of 12-well transwell co-culture system at a density of 5 × 10^5^ cells/mL, while RAW264.7 cells were seeded in the upper chamber at 5 × 10^5^ cells/mL. Then the cells were stimulated with 200 ng/mL LPS and various concentrations of ILDL and incubated for 48 h total. The MODE-K cell viability was detected by CCK-8 and apoptotic cells were detected flow cytometry.

### Macrophage efferocytosis detection

Incubation with various concentrations of ILDL was conducted for 24 h on RAW264.7 cells seeded on 12-well plates. MODE-K cells were stained by CM-DIL and then induced by apoptosis-positive control solution (Multi Sciences, Hangzhou, China) on ice for 30 min according to the directions. Apoptotic MODE-K cells were collected and counted. Add apoptotic MODE-K cells to the RAW264.7 cells in the 12-well plate. After co-incubating for 2 h and 16 h, removed the apoptotic MODE-K cells and collected RAW264.7 cells, Cells stained with anti-F4/80 antibody and detected by flow cytometry to calculate the efferocytosis index (efferocytosis index = Positive macrophages / all macrophages).

### Silencing of LXRα and its impact on the anti-inflammatory effect of ILDL

RAW264.7 cells were seeded in 12-well plates at a density of 1 × 10^5^ cells/mL. To enhance transfection efficiency, cells were pretreated with Nucleic Acid Transfection Enhancer (NATE™, InvivoGen, #lyec-nate) at a 1X working concentration (diluted from 100X stock) for 30 min with gentle mixing. LXRα silencing was performed using RNAiMAX transfection reagent (Thermo Fisher Scientific, #13,778,150) with specific siRNA sequences (see Supplementary Table S1) for 48 h. Following transfection, cells were stimulated with LPS (200 ng/mL) and ILDL for an additional 12 h.

### Experimental animals

#### At 8 weeks of age, male C57BL/6 J mice were purchased from the Experimental

Animal Center of Hangzhou Medical College (Hangzhou, China). Mice were housed in a barrier system with a clean environment at 20–25 °C under 50–60% humidity and 12 h of light. In this study, Animals were cared for humanely according to Hangzhou Medical College's institutional animal ethics guidelines (Approval NO. ZJCLA-IACUC-20020147).

### Anti-ulcerative colitis action of ILDL

#### Disease models and administration of ILDL

Forty-two male mice were randomly divided into six groups after quarantine and adaptation: the normal control group (NC), the model control group (MC), the positive control group (SASP), and ILDL-L (2 mg/kg), ILDL-M (6 mg/kg), ILDL-H (20 mg/kg) groups. All five groups, with the exception of NC, received 2.5% DSS dissolved in autoclaved water for 9 days, changing the solution every two days. ILDL (2 mg/kg, 6 mg/kg, and 20 mg/kg), SASP (200 mg/kg), or equivalent 0.5% CMC-Na solution was administered by gavage for 9 days. All mice were sacrificed after anesthetized by pentobarbital sodium on the 10th day.

### Body weight, stool consistency, and stool occult blood

Body weight and fecal characters of mice in each group were daily observed and recorded throughout the experiment. The conditions of weight loss were scored as follows: no loss scored 0, 1–5% scored 1, 5–10% scored 2, 10–20% scored 3, > 20% scored 4. The conditions of stool consistency were scored as follows: normal stool (dark brown, hard ellipsoid) was scored 0, loose scored 1, watery diarrhea scored 2, slimy diarrhea, little blood scored 3, severe watery diarrhea with blood scored 4. The conditions of stool occult blood were scored as follows: no blood scored 0, presence of blood scored 2, gross bleeding scored 4. The above three scores were added to the disease activity index (DAI) [22].

### Colonic morphology, length, and histopathological examination

The mice were fasted for 12 h after the last administration, but water was given. The mice were sacrificed after weighting. The entire colon was removed to measure its morphology and length. Then the colonic tissue was washed with 0.9% sodium chloride solution and then placed in 10% formalin. Hematoxylin and Eosin (H&E), Periodic Acid-Schiff (PAS), and Masson staining were performed and observed under a microscope.

### Real-time quantitative PCR

The colon was quickly removed and washed by pre-cooled saline solution, then immediately flash freeze in liquid nitrogen for 30 min and stored at – 80 ℃. Total RNA was extracted from colon and reverse transcribed to cDNA using PrimeScript™ RT Master Mix (TAKARA BIO INC.). The expression levels of target genes were measured by Real-time quantitative PCR (qPCR). Amplification conditions were as follows: initial denaturation at 95 ˚C for 30 s, then forty cycles of 95 ˚C for 5 s, and then 60 ˚C for 30 s. A single dissociation peak was detected in each reaction by the dissociation curve. The expression of each gene was normalized to the GAPDH gene on the base of the 2^−ΔΔCT^ algorithm. The primers of target genes are listed in the Supplementary Table S2 and were obtained from Ykang Biotech (Hangzhou, China).

### Western blotting

Colon tissue was cut and resuspended in 1 mL ice-cold RIPA lysis buffer and crushed by a tissue shredder. Cell samples were lysed with RIPA buffer with protease inhibitors. Then centrifuged for 15 min at 12,000 × g at 4℃. Total protein concentration was determined by the BCA Protein Assay Kit (Thermofisher, MA, USA). Then, the samples were separated by sodium dodecyl sulfate–polyacrylamide gel electrophoresis (SDS-PAGE) and transferred onto polyvinylidene fluoride (PVDF) membranes. Membranes were blocked with quick block reagent (beyotime, shanghai, China) for 10 min at room temperature, followed by incubation with primary antibody overnight at 4 °C and horseradish peroxidase-conjugated second antibody (HUABIO, Hangzhou, China) for an hour at room temperature. Immunoreactive proteins were visualized with an enhanced chemiluminescence (ECL) Western blot detection system. Western blot quantifications were analyzed by Image J software.

### Immunohistochemistry

CD86 and CD206 were detected by immunohistochemistry of colon tissue using an immunohistochemistry kit (abcam, MA, USA). In brief, paraffin-embedded slides were deparaffinized, rehydrated, and washed in PBS. Followed by 0.3% hydrogen peroxide treatment and 30 min of blocking at 37 °C. Incubation was performed at 4 °C overnight with primary antibodies. FITC or CY5-labeled secondary antibodies were incubated at room temperature for 1 h. After washing the section by TBST, DAPI was added to stain the nucleus for 5 min. Finally, the sections were washed and an anti-fluorescence quencher was added, and observed under fluorescence microscope.

### Drug affinity responsible target stability (DARTS) technology

RAW264.7 cells were lysed on ice for 10 min, and centrifuge for 10 min at 18,000 × g at 4 °C. Cell supernatant was collected and added an appropriate volume of 10 × TNC buffer to make a final concentration of 1 × TNC buffer in the lysate. The protein concentration of the cell lysate was detected and regulated to 2.5 mg/mL. Other procedures were done according to the literature [23]. After DARTS, perform SDS-PAGE, transfer the proteins to the PVDF membrane, and blot the membrane with LXRα antibody. Immunoreactive proteins were visualized with a western blot detection system.

### Molecular docking

LXRα protein information and its three-dimensional structure (PDB ID:3IPQ) were downloaded from the RCSB database [24]. Isolinderalactone (CID: 5,318,587) structure was downloaded from the PubChem database. AutoDock Tools 1.5.6 and MOPAC program were used to optimize the molecular structure for subsequent molecular docking [25, 26]. According to the literature, the center coordinate of the docking box is set as the potentially active pocket center and the whole protein active pocket is wrapped. The result of molecular docking may have unreasonable atomic contact in space structure. The energy optimization method was used to release these forces and make them a more stable structure [27].

### Statistical analysis

Drawing figures and statistical analyses were performed by GraphPad Prism software. Quantitative results are presented as means ± standard deviations (SD). Comparisons between two groups were analyzed by Student’s t-test. Statistical differences were assessed by one-way analysis of variance (ANOVA) followed by a post LSD (homogeneity) or Games-Howell (Heterogeneity) when comparing more than two groups. *P*-values less than 0.05 were considered statistically significant.

## Results

### Isolinderalactone significantly inhibits M1 polarization of macrophages and exhibits excellent anti-inflammatory activity

**Fig.1:**
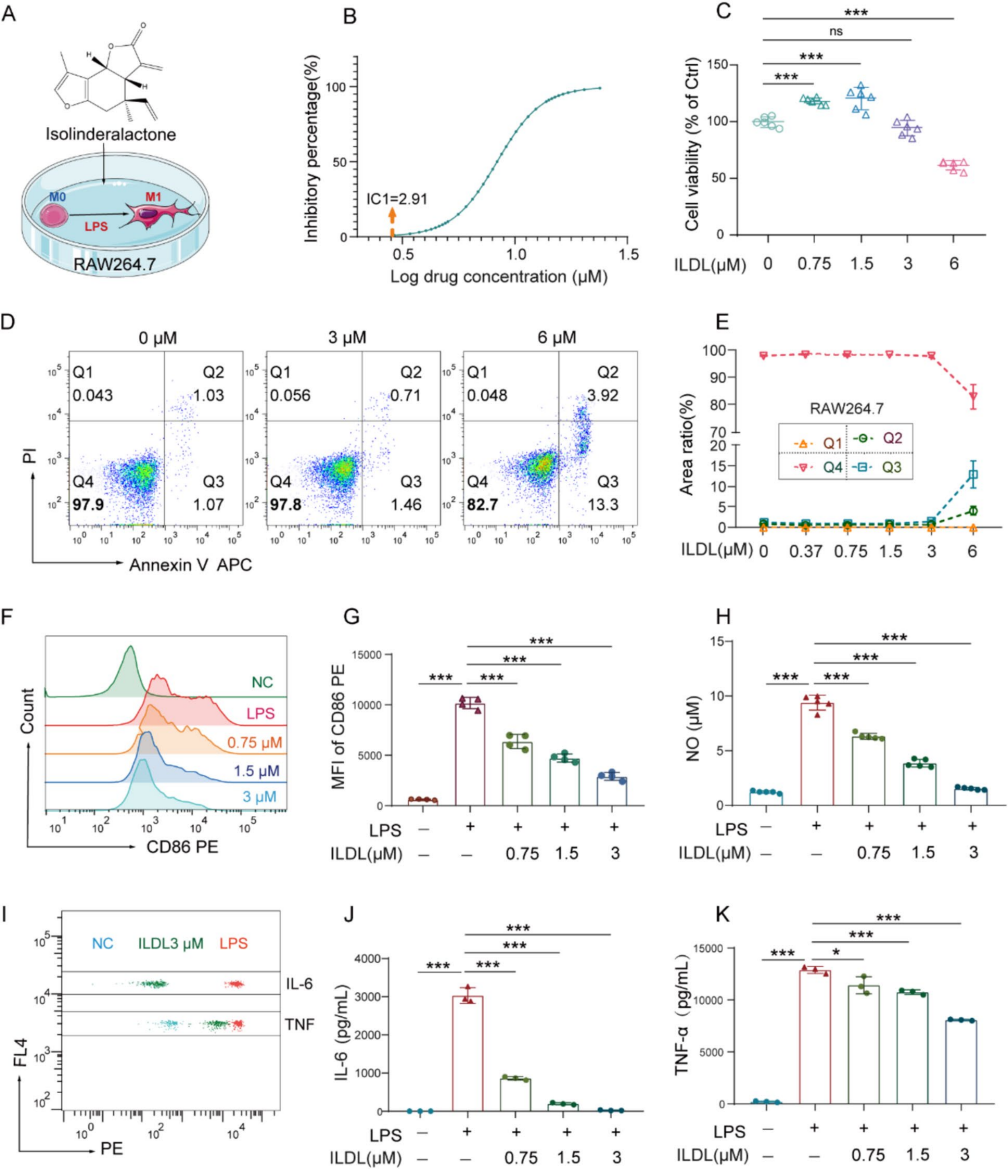
Isolinderalactone (ILDL) effectively inhibits macrophage M1 polarization and the production of inflammatory mediators. **A** Schematic of the experimental strategy in vitro. **B** Inhibition curve of ILDL on RAW264.7cells. **C** Cytotoxicity of RAW264.7cells after 48 h of ILDL treatment (n = 6). **D**, **E** The cytotoxicity of ILDL was verified by Annexin V-APC/PI apoptosis assay (n = 3). **F** Histogram of macrophage M1 polarization detected by flow cytometry. **G** Effect of ILDL treatment on mean fluorescence intensity (MFI) of CD86 (n = 4). **H** Effect of ILDL treatment on Nitric oxide (NO) in RAW264.7 cells supernatants (n = 5). **I–K** Detection and statistics of IL-6 and TNF-α expression in RAW264.7 cells supernatants by Cytometric Bead Array (n = 3). Data shown as mean ± SD (*P < 0.05, ***P < 0.001; ns not significant)

LPS-induced macrophage inflammation model was used to evaluate the anti-inflammatory effect of ILDL in vitro (Fig. 1A). Initially, we explored the cytotoxicity of different concentrations of ILDL on RAW264.7 cells using the CCK-8 assay. The fitted curve of the drug’s inhibitory effect suggested that the maximum noncytotoxic concentration of ILDL was about 2.91 μmol/L (Fig. 1B). Then, we further employed the annexin V/ PI apoptosis assay to confirm the cytotoxicity of different ILDL concentrations on cell survival and apoptosis. The results showed that at 6 μmol/L, ILDL exhibited cytotoxicity toward RAW264.7 cells, but no cytotoxicity was observed at 3 μmol/L in both CCK-8 and apoptotic assays. (Fig. 1D, E). Therefore, we selected 3 μmol/L as a high dose for anti-inflammatory treatment in vitro in this study.

We induced the M1 polarization of RAW264.7 cells from M0 to M1 using LPS for 48 h. Flow cytometry results demonstrated that LPS significantly induced M1 differentiation and the production of inflammatory mediators, such as NO, IL-6, and TNF compared with the normal control group. The M1 phenotypic marker CD86 expression and NO content were significantly reduced in the ILDL intervention group versus the LPS-induced group (Fig. 1F–H). Moreover, the flow cytometry Cytometric Bead Array showed significant reductions in the production of IL-6 and TNF in the ILDL intervention group (Fig. 1I–K). Furthermore, we also screened the anti-inflammatory activity of other characteristic monomer components of *Lindera aggregata*, such as lindenenyl acetate, linderane, linderalactone, and so on (Fig. S1A-J). We found that ILDL has a superior anti-inflammatory activity among many *Lindera aggregata* components (Supplementary Fig. S1K). These results suggested that ILDL could dose-dependently inhibit LPS-induced M1 polarization and the production of inflammatory mediators, but its mechanism remains unclear and needs further exploration.

### Transcriptomics reveal potential involvement of Liver X receptor (LXR)-mediated signaling pathways in the anti-inflammatory effect of ILDL

**Fig.2:**
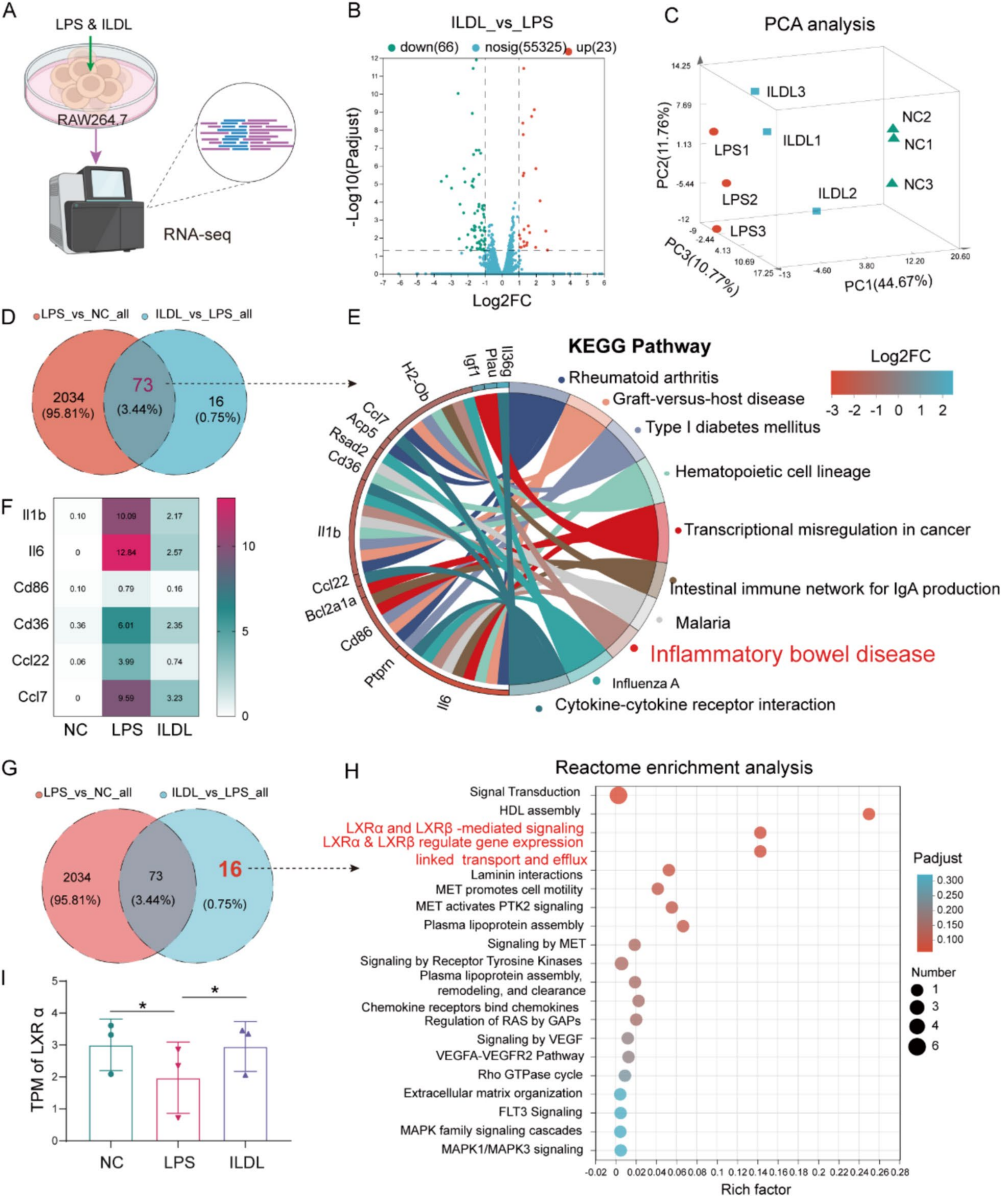
Transcriptomics reveal potential involvement of Liver X receptor (LXR)-mediated signaling pathways in the anti-inflammatory effect of ILDL.** A** Schematic representation of the RAW 264.7cells transcriptome. **B** RNA-seq volcano plot of genes in RAW264.7 cells between the ILDL group and LPS group. **C** Principal component analysis of the NC, LPS, and ILDL groups (n = 3). **D**, **G** Venn diagram showing the similarities and differences between the LPS&NC and ILDL&LPS gene sets. **E** KEGG signaling pathway enrichment analysis of the 73 common genes between the LPS&NC and ILDL&LPS gene sets. **F** Heatmap showing the relative mRNA levels of genes associated with cytokines among the NC, LPS, and ILDL groups. **G-I** Effect of ILDL treatment on IL-1β, IL-6, and TNF-α mRNA expression in RAW264.7 cells (n = 3). **H** Reactome enrichment analysis of the 16 unique genes to ILDL&LPS gene set. **I** TPM of LXRα among the NC, LPS, and ILDL groups (n = 3). Data shown as mean ± SD (*P < 0.05, ***P < 0.001)

Transcriptomics was used to further confirm the anti-inflammatory effect of ILDL and explore its possible mechanism (Fig. 2A). The volcano map of differential expression genes (DEGs) showed 89 statistically significant DEGs (66 down-regulated and 23 up-regulated) between the ILDL and LPS groups (Fig. 2B). PCA analyse showed that the NC, LPS, and ILDL groups were classified, indicating significant differences among the three groups (Fig. 2C). Compared with the LPS group, the samples of the ILDL group were closer to the NC group, suggesting that the gene expression of the ILDL group and the NC group was more similar, and indicating that ILDL could significantly improve LPS-induced gene expression.

Venn diagram analysis revealed 73 common genes between the LPS&NC differential gene set and the ILDL&LPS differential gene set, along with 16 unique genes associated with ILDL (Fig. 2D). KEGG signaling pathway enrichment analysis showed that these 73 genes were significantly enriched in pathways related to rheumatic arthritis, inflammatory bowel disease, and so on (Fig. 2E), suggesting that ILDL may have an anti-IBD effect. Heat map analysis of genes related to Top10 enrichment signaling pathways demonstrated a significant reduction in mRNA content of IL-1β, IL-6, and CD86 with ILDL treatment (Fig. 2F). Furthermore, we performed a functional enrichment analysis of 16 genes unique to ILDL. Reactome enrichment analysis showed that the 16 genes were significantly enriched in LXRα (also known as NR1H3) and LXRβ (also known as NR1H2)-mediated signaling, LXRα & LXRβ regulate gene expression linked to transport and efflux and HDL assembly (Fig. 2G-I). In summary, the above transcriptomic results confirmed that ILDL possesses potent anti-inflammatory activity and suggested that its anti-inflammatory mechanism may be closely related to the LXR-mediated signaling pathway, which warrants further verification.

### ILDL interacts with LXRα and activates LXRα mediated signaling

**Fig. 3:**
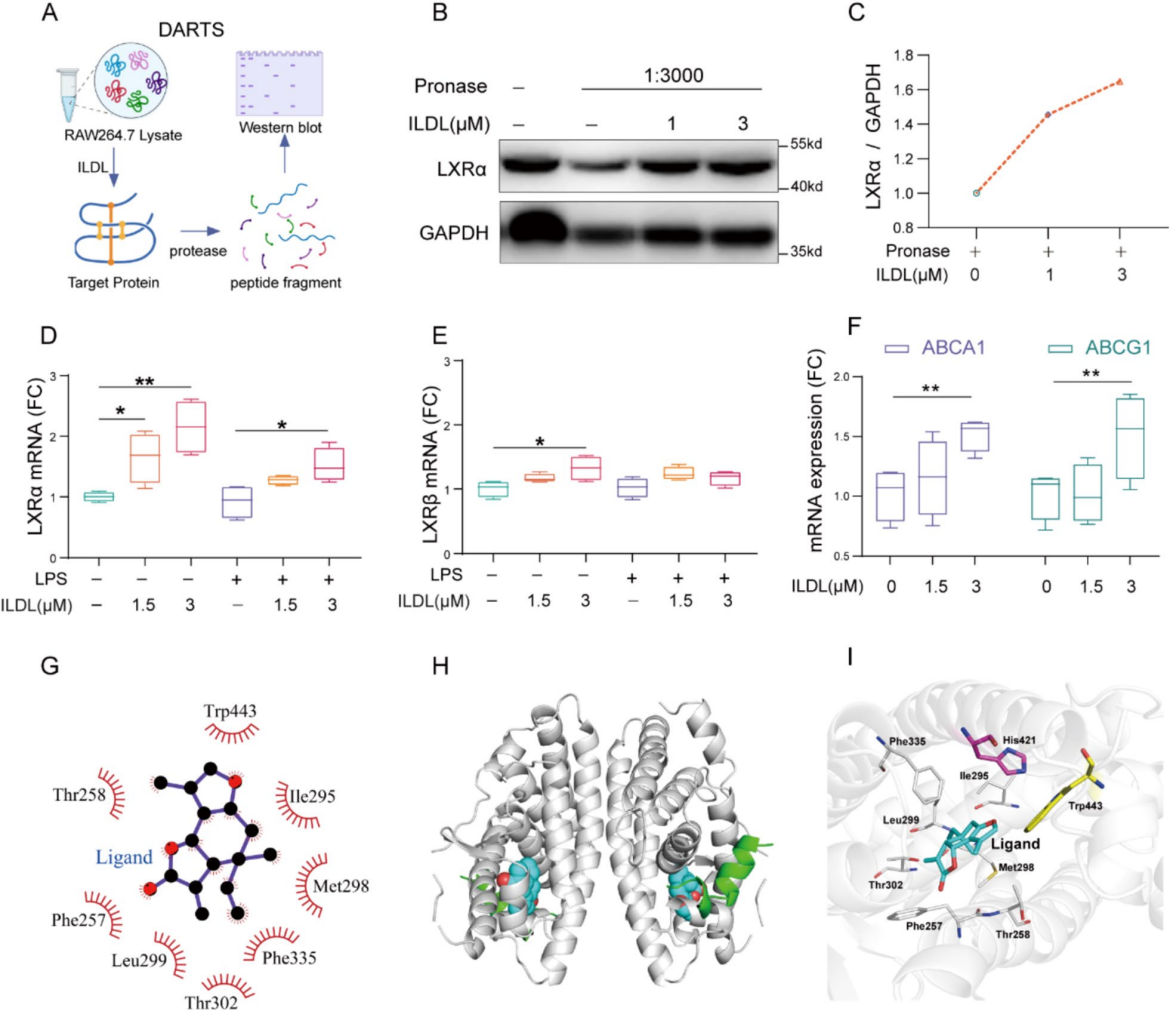
ILDL combines with LXRα and regulates LXRα and its target gene expression. **A** Schematic of Drug affinity responsible target stability.** B**, **C** Western blot and densitometry analysis of LXRα. **D**, **E** Effect of ILDL treatment on LXRα and LXRβ mRNA expression with or without LPS (n = 3). **F** Effect of ILDL treatment on ABCG1 and ABCA1 mRNA expression without LPS (n = 3). **G** Two-dimensional binding pattern of ILDL and LXRα. **H** Position of ILDL in the three-dimensional structure of the LXRα protein. **I** Three-dimensional binding pattern of ILDL and LXRα. Data shown as mean ± SD (*P < 0.05, **P < 0.01, ***P < 0.001; ns not significant)

According to transcriptomic results, drug affinity responsible target stability (DARTS) was adopted to verify the binding effect between ILDL and LXRα (Fig. 3A). Western blot results showed that compared with samples without ILDL intervention, the amount of demonstrated a dose-dependent increase in LXRα protein levels upon ILDL addition (Fig. 3B, C), confirming ILDL’s ability to significantly reduce LXRα degradation by pronase through binding. Literature reports that LXR agonists induce LXR and its target genes, such as ABCA1 and ABCG1mRNA expression upon LXR activation [28]. Thus, we assessed the expression of genes related to LXR signaling under normal and LPS induction conditions. We observed that under normal conditions, both 1.5 and 3 μmol/L ILDL significantly increased LXRα mRNA expression, with only 3 μmol/L ILDL inducing LXRβ mRNA expression. Similarly, under LPS-induced conditions, 3 μmol/L ILDL significantly increased LXRα mRNA but had no significant effect on LXRβ mRNA (Fig. 3D, E), indicating a greater effect of ILDL on LXRα than LXRβ. Moreover, further analysis revealed that ILDL significantly upregulated the expression of LXRα target genes, ABCA1 and ABCG1 (Fig. 3F), suggesting activation of the LXRα pathway by ILDL. Furthermore, we used AutoDock Tools and the molecular docking method to analyze the interaction between ILDL and the LXRα protein. The findings revealed a binding energy of -8.106 kcal/mol and a predicted K_d_ value of 1.13 × 10^–6^ mol/L, indicating a strong binding between ILDL and the LXRα protein. Further analysis revealed that ILDL binds to a cavity consisting of Phe257, Thr258, Ile295, Met298, Leu299, Thr302, Phe335, and Trp443 amino acids through hydrophobic interactions, thereby promoting stable binding between ILDL and LXRα protein (Fig. 3G-I).

### ILDL regulates anti-inflammatory and other pharmacological effects mediated by LXRα activation

**Fig.4:**
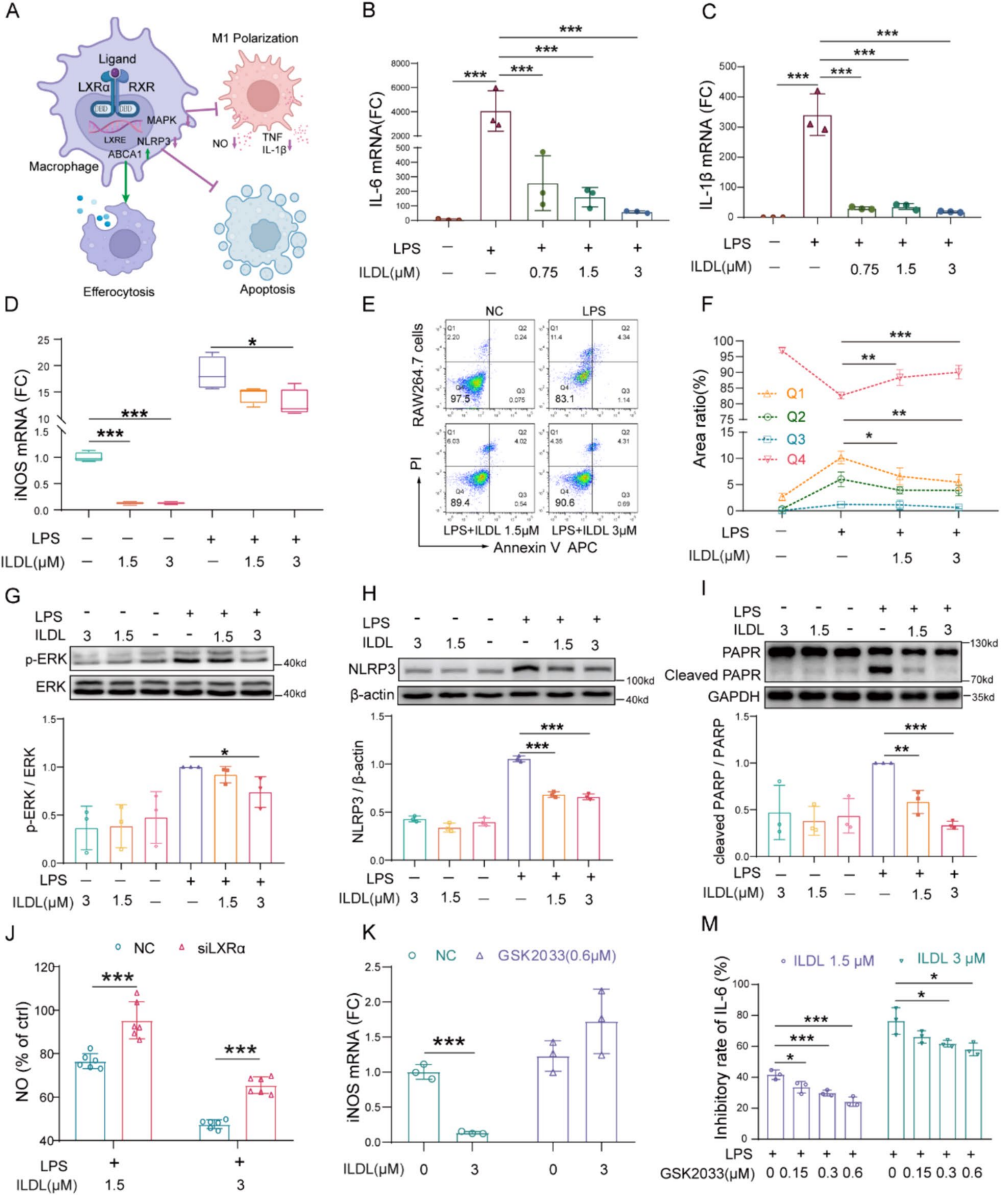
ILDL regulates LXRα mediated signaling pathway and effects in macrophage. **A** Schematic of the effect of LXRα activation in macrophage.** B-D** Statistics of IL-6, IL-1β, and iNOS mRNA expression with LPS and ILDL intervention.** E**, **F** Flow cytometry and statistics of ILDL protected macrophage apoptosis (n = 3). **G-I** Western blot and densitometry analysis of Phospho-ERK/ERK, NLRP3, and cleaved PARP/PARP after 48 h of ILDL treatment with or without LPS (n = 3).** J** The inhibition of ILDL on NO in culture supernatants after silencing LXRα (n = 6). **K**, **M** The inhibition of ILDL on IL-6 in culture supernatants and relative fold change of iNOS mRNA in RAW264.7 cells with or without LXRα inhibitor GSK2033 (n = 3). Data shown as mean ± SD (*P < 0.05, **P < 0.01, ***P < 0.001; ns not significant)

Activation of LXRα signaling pathway can not only regulate M1 polarization and the production of pro-inflammatory mediators by inhibiting MAPK and other pathways, but also prevent macrophage apoptosis by inhibiting NLRP3 activation and PARP shearing and promote macrophage efferocytosis by increasing ABCA1 mediated cholesterol efflux (Fig. 4A). On one hand, we found that ILDL can significantly inhibit the phosphorylation of ERK and decrease the mRNA expression of IL-6, IL-1β and iNOS induced by LPS (Fig. 4B-D, G). On the other hand, ILDL can significantly reduce the apoptosis of macrophages and protect the survival of macrophages under LPS stimulation (Fig. 4E, F). Western blot results confirmed that ILDL could prevent macrophage apoptosis by inhibiting NLRP3 activation and PARP shearing (Fig. 4H, I). The above results indicates that ILDL can activate the LXRα pathway to inhibit NLRP3 activation, MAPK signal transduction, and LPS-induced DNA damage, which was consistent with the reported effect of LXR agonists.

Subsequently, using siRNA technology to silence LXRα, we observed that the effect of 1.5 μmol/L and 3 μmol/L ILDL in reducing NO was significantly decreased compared to cells with normal LXRα expression (Fig. 4J). Moreover, we further used the LXRα inhibitor GSK2033 to inhibit LXRα activity. Our findings revealed that ILDL significantly reduced iNOS mRNA expression under normal conditions, a reduction that was entirely abolished when GSK2033 inhibited LXRα activity (Fig. 4K). Similarly, GSK2033 at concentrations of 0.15 μmol/L, 0.3 μmol/L, and 0.6 μmol/L could dose-dependently attenuate the inhibitory effect of ILDL on IL-6 production (Fig. 4M). These results confirm that ILDL inhibits the production of inflammatory mediators by activating the LXRα pathway.

### ILDL protects intestinal epithelial cells by regulating macrophage polarization and efferocytosis

**Fig.5:**
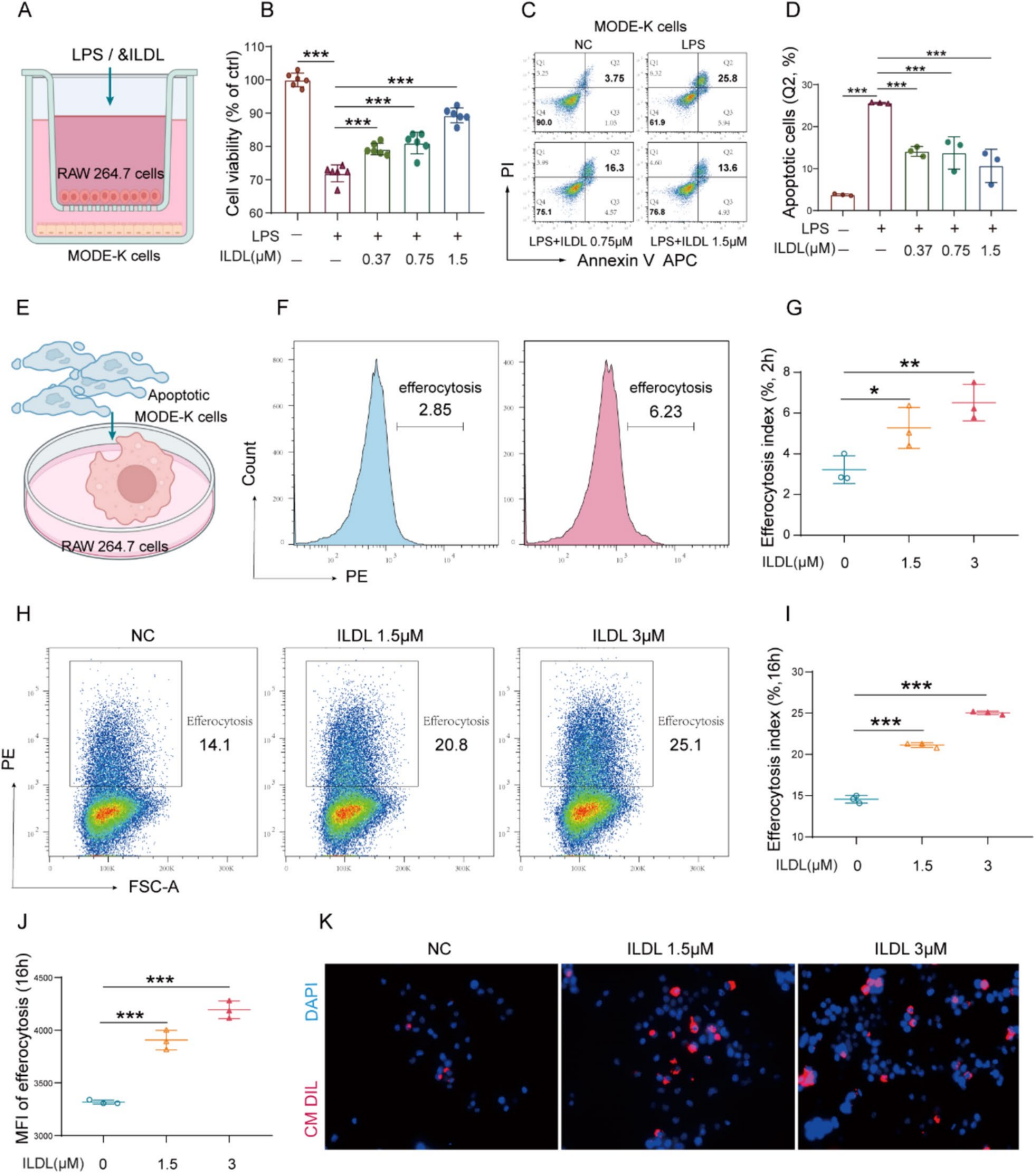
ILDL protects intestinal epithelial cells by inhibiting macrophage M1 polarization and enhancing efferocytosis capacity.** A** Schematic of co-culture system of intestinal epithelial cells and macrophages.** B** Cell viability of MODE-K cells after 48 h of ILDL treatment (n = 6). **C**, **D** Detection and statistics of apoptosis of MODE-K cells in co-culture experiments after 48 h of ILDL treatment (n = 3). **E** Schematic of the efferocytosis experimental strategy.** F**,** G** Detection and statistics of efferocytosis index after added apoptotic MODE-K cells to RAW264.7 cells for 2 h (n = 3).** H**-**J** Detection and statistics of efferocytosis index and MFI of efferocytosis after added apoptotic MODE-K cells to RAW264.7 cells for 16 h (n = 3).** K** Fluorescence images of efferocytosis after added apoptotic MODE-K cells to RAW264.7 cells for 16 h. Data shown as mean ± SD (*P < 0.05, **P < 0.01, ***P < 0.001; ns not significant)

The activation of LXRα in macrophages not only mediates the anti-inflammatory effect but also promotes macrophage-mediated efferocytosis. To detect the protective effect of ILDL on intestinal epithelial cells, mouse macrophages (RAW264.7) were co-cultured with mouse intestinal epithelial cells (MODE-K cells) using a transwell co-culture system in vitro. We used LPS to induce macrophages to secrete pro-inflammatory mediators to damage intestinal epithelial cells to simulate intestinal inflammation (Fig. 5A). Initially, we confirmed that ILDL at a concentration of 1.5 μmol/L was not cytotoxic to MODE-K cells through CCK-8 and annexin V / PI apoptosis assay, which could be used as a high dose for co-culture tests. Compared with the normal co-culture group, LPS induction for 48 h significantly increased the secretion of pro-inflammatory mediators by upper chamber RAW264.7 cells and induced damage to lower chamber MODE-K cells, resulting in a significant decrease in epithelial cell viability and increased cell apoptosis. Compared with LPS control group, treatment with ILDL at concentrations of 0.37 μmol/L, 0.75 μmol/L, and 1.5 μmol/L significantly increased the cell viability of MODE-K cells (Fig. 5B), suggesting that ILDL can protect intestinal epithelial cell damage in inflammatory state.

Moreover, the results of apoptosis assay showed that ILDL could effectively reduce the apoptosis rate and increase the cell survival rate (Fig. 5C, D). These results suggest that ILDL may alleviate damage to intestinal epithelial cells by inhibiting macrophage polarization.

Furthermore, we simulated the apoptosis and clearance of a large number of intestinal epithelial cells in the UC state. We induced apoptosis of intestinal epithelial cells using apoptosis inducers and then added them to RAW264.7 cells for co-feeding and detection of efferocytosis in vitro (Fig. 5E). Macrophages can recognize apoptotic intestinal epithelial cells and phagocytic them to initiate efferocytosis. Flow cytometry showed that after co-culturing apoptotic MODE-K cells with RAW264.7 for 2 h, RAW264.7 was able to phagocytize apoptotic epithelial cells, and the efferocytosis index of RAW264.7 was about 2.85%. However, ILDL at concentrations of 1.5 μmol/L and 3 μmol/L significantly increased the efferocytosis index to about 5% and 6%, respectively (Fig. 5F, G). After co-culture for 16 h, the efferocytosis index increased to 14.1%. ILDL at concentrations of 1.5 μmol/L and 3 μmol/L not only significantly increased efferocytosis index to about 20% and 25%, respectively (Fig. 5H, I), but also significantly increased the average fluorescence intensity of RAW264.7 cells (Fig. 5J, K), suggesting that ILDL could not only increase the number of macrophages initiating efferocytosis but also enhance the phagocytosis of apoptotic epithelial cells by macrophages, demonstrating a significant pharmacological in promoting efferocytosis.

### Isolinderalactone effectively improves symptoms and tissue lesions in acute DSS-induced UC mice

**Fig.6:**
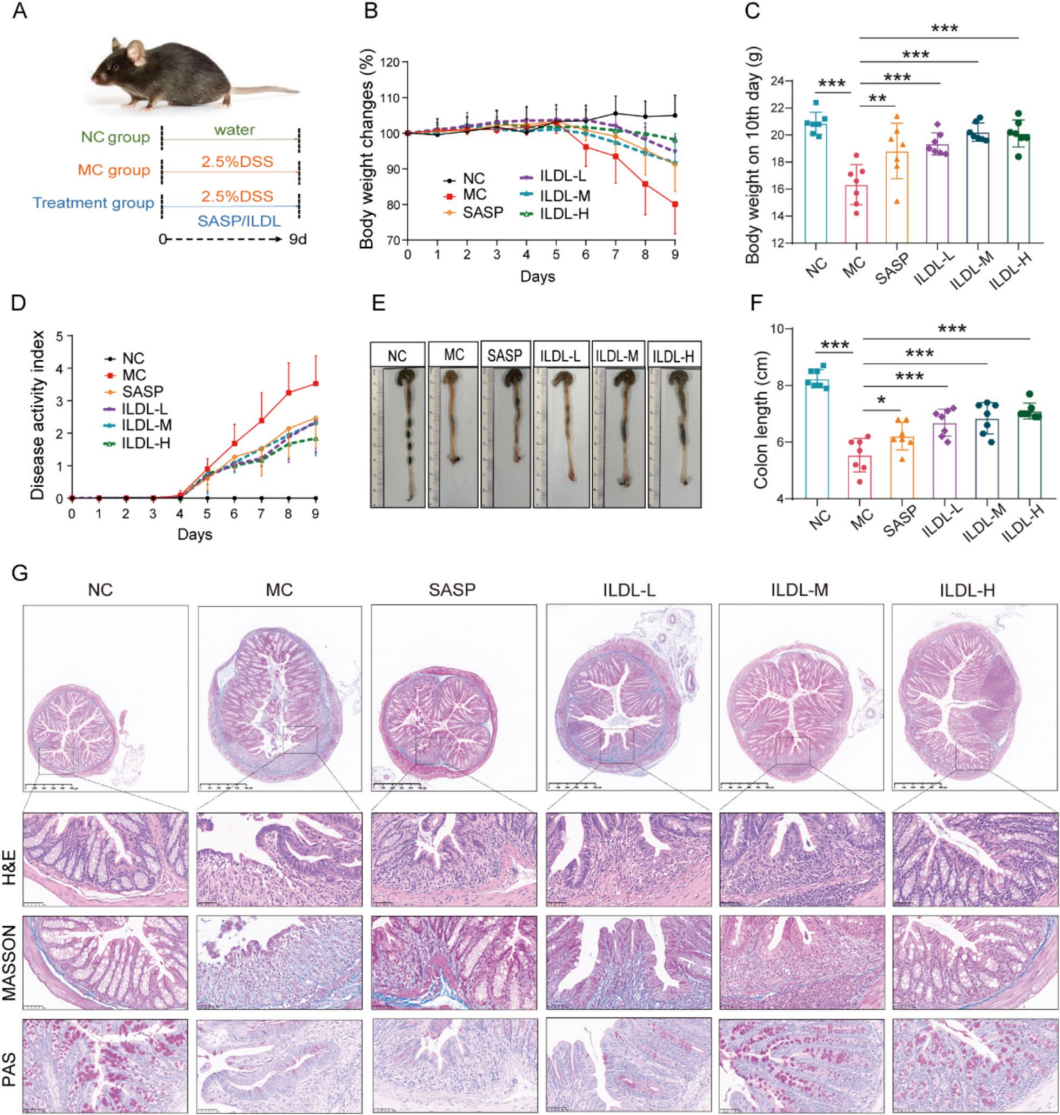
ILDL effectively improves symptoms and tissue lesions in acute DSS-induced UC Mice.** A** Schematic of the experimental strategy. **B** Body weight change (n = 7). **C** Statistics of body weight on 10th day (n = 7). **D** Chart of disease activity index trends (n = 7). **E** The gross morphology and length of colon. **F** Statists of the colon length (n = 7). **G** Representative microscopic pictures of colon H&E, Masson and Periodic Acid-Schiff staining. Data shown as mean ± SD (*P < 0.05, **P < 0.01, ***P < 0.001; ns not significant)

To evaluate the anti-UC efficacy of ILDL, a classical acute ulcerative colitis model induced by DSS was used (Fig. 6A). Free drinking of a 2.5% DSS water solution successfully induced UC-like symptoms, including weight loss and bloody stools in mice, confirming model duplication. Compared with the model control group, mice treated with sulfasalazine (SASP, 200 mg/kg) or ILDL (2, 6, and 20 mg/kg) exhibited reduced weight loss and disease activity index scores, increased colon tissue length, and observable alleviation of degree of colonic morphology and pathological changes (Fig. 6B-F). Histological analysis via H&E staining revealed severe damage to colonic epithelial cells and large ulcer foci with extensive immune cell infiltration in the model control group. Masson staining indicated increased fibrosis in the colonic tissues of the model control group, particularly in the intrinsic layer of the ulcer foci. PAS staining demonstrated reduced mucus secretion by colonic epithelial cells in the model control group. However, in ILDL dose groups, colonic epithelial cell damage, ulceration, and fibrosis were significantly improved, with increased mucus secretion in the colon tissue of ILDL (Fig. 6G). These findings suggest that ILDL effectively improves UC disease symptoms and tissue lesions, exhibiting promising anti-UC effects.

### Transcriptomics further confirmed the anti-UC pharmacological effect of ILDL

**Fig.7:**
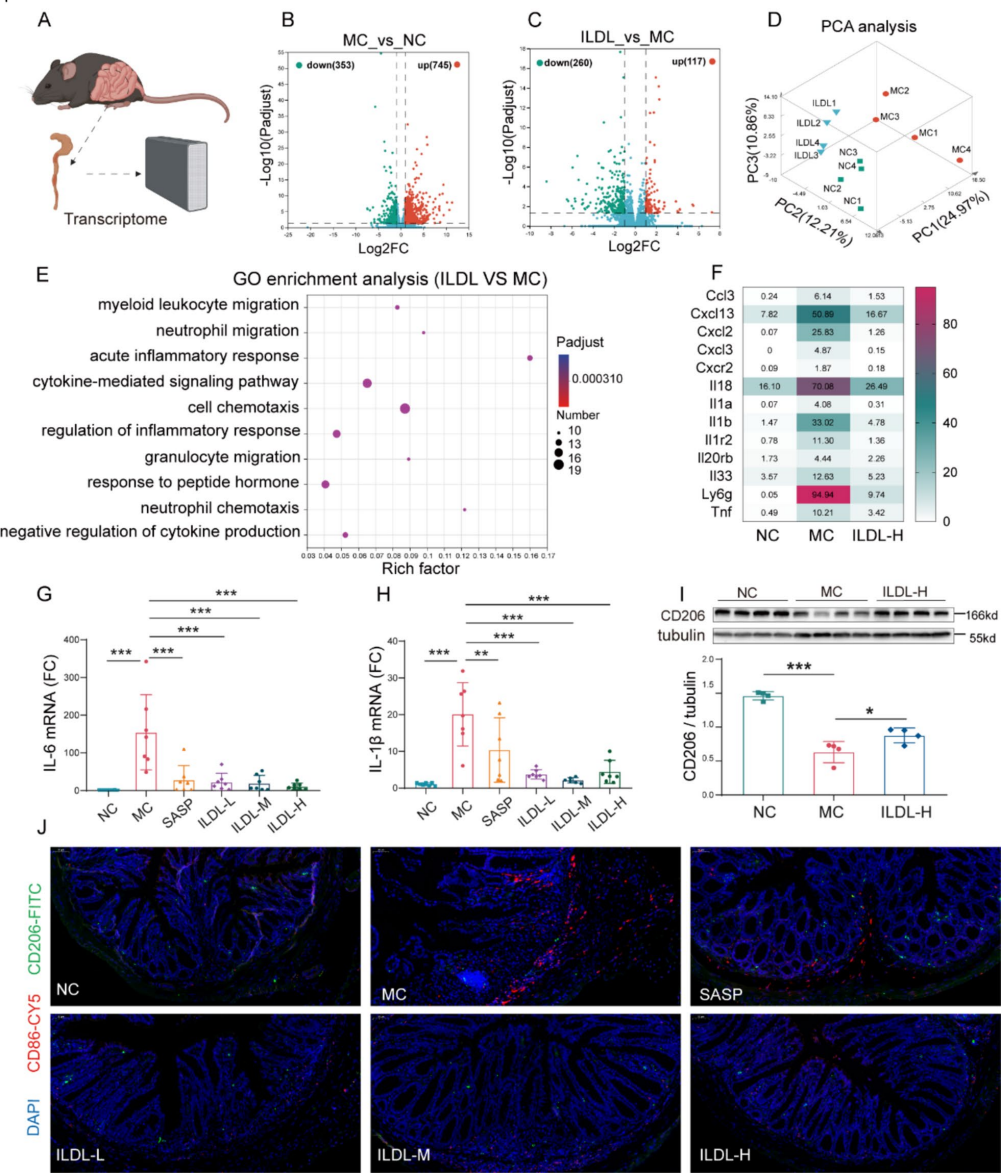
Transcriptomics further confirmed that the anti-UC effect of ILDL is related to its anti-inflammatory activity.** A** Schematic representation of the tissue transcriptome. **B** Colonic RNA-seq volcano plot of genes between the MC group and the NC group. **C** Colonic RNA-seq volcano plot of genes between the ILDL group and the MC group. **D** Principal component analysis of the NC, MC, and ILDL groups (n = 4). **E** GO enrichment analysis of DEGs between ILDL and MC groups. **F** RNA-seq heatmap showing the differential express genes (DEGs) associated with chemokines and cytokines among the NC, MC, and ILDL groups (n = 4) **G**,** H** Effect of ILDL treatment on IL-6, and IL-1β mRNA expression in the colon (n = 7).** I** Western blot and densitometry analysis of CD206 protein expression in the colon (n = 4). **J** Immunohistochemical image of CD86 and CD206 in the colon tissue. Data shown as mean ± SD (***P < 0.001; ns not significant)

To further confirm the anti-UC pharmacological effect of ILDL, transcriptomics was employed to assess gene expression changes in colon tissues across experimental groups. The volcano plot of DEGs revealed 1098 statistically significant DEGs (353 down-regulated and 745 up-regulated genes) between the MC and NC groups, and 377 statistically significant DEGs (260 down-regulated and 117 up-regulated) between ILDL and MC groups (Fig. 7B, C). Principal component analysis was used to analyze differences and similarities in colon tissue among the groups. As shown in Fig. 7D, clear delineation was observed among the NC, MC, and ILDL subgroups, indicating significant differences among the three groups. Notably, compared with the MC group, samples from the ILDL group exhibited closer proximity to those of the NC group, suggesting greater similarity in gene expression between the ILDL and NC groups, consistent with the protective effect demonstrated in animal experiments.

GO enrichment analysis demonstrated that DEGs between the ILDL and MC groups were significantly enriched in inflammatory pathways, including myeloid leukocyte migration, acute inflammatory response, and negative regulation of cytokine production (Fig. 7E). Macrophages, as crucial innate immune cells, exhibit remarkable plasticity and multifunctionality, playing a central role in acute UC phase by secreting a variety of chemokines and cytokines. Heat map analysis of cytokines and chemokines gene expression showed decreased expression of IL-1β, IL18, and cxcl13 in the ILDL group to the model control group (Fig. 7F). qPCR confirmed ILDL's ability to significantly reduce mRNA expression of IL-1β and IL-6 (Fig. 7G, H), indicating its potential to inhibit immune cell chemotactic aggregation and effectively reduce inflammation. Immunohistochemical and western blot results of colon showed that macrophage M1 marker CD86 was significantly increased and M2 marker CD206 was significantly decreased in the model control group. Compared with the model control group, the M1 marker CD86 was significantly decreased and M2 marker CD206 was increased significantly in ILDL intervention groups. These findings indicate that ILDL effectively inhibits M1 polarization of macrophages both in vivo and in vitro, improves UC disease symptoms and tissue lesions, exhibiting promising anti-UC effects.

## Discussion

UC is a common inflammatory gastrointestinal disease closely associated with macrophage polarization imbalance and defective efferocytosis. In our previous research, we observed that the ethanol extract of *Lindera aggregata* not only significantly inhibits the production of inflammatory mediators but also improves symptoms and histopathological changes in rat and mouse UC models. However, the specific anti-inflammatory and anti-UC active substances of *Lindera aggregata* have remained unidentified. Therefore, we conducted a screening and comparative study on the anti-inflammatory activity of known characteristic components. Our findings revealed that ILDL dose-dependently inhibit macrophage M1 polarization and inflammatory mediator production, exhibiting the best anti-inflammatory activity and potency among various characteristic components of *Lindera aggregata*. However, its mechanism remains unclear and needs further exploration.

Initially, we investigated ILDL as a kinase inhibitor and found that short-term intervention of ILDL did not significantly affect the phosphorylation of P65 in the nuclear factor kappa B (NF-κB) pathway and ERK in the mitogen-activated protein kinase (MAPK) pathway, suggesting that ILDL does not directly act as a kinase inhibitor. Therefore, we comprehensively explored ILDL's anti-inflammatory mechanism using transcriptomics. The Venn diagram revealed 73 common genes between the LPS&NC differential gene set and the ILDL&LPS differential gene set, with 16 unique genes attributed to ILDL. KEGG signaling pathway enrichment analysis demonstrated significant enrichment of the 73 genes in inflammatory bowel disease, consistent with the anti-UC pharmacological effect observed in animal experiments. Importantly, the 16 genes unique in the ILDL intervention group were significantly enriched in LXRα and LXRβ-mediated signaling, LXRα & LXRβregulate gene expression linked transport and efflux and HDL assembly, indicating that the anti-inflammatory mechanism of ILDL may be closely related to the LXR-mediated signaling pathway.

The liver X receptor functions as a transcriptional regulator, forming a heterodimer with the retinoic X receptor. The nuclear localization of the LXR has been associated with transcription activity, with two isoforms identified, α and β. Initially, we evaluated the effect of ILDL on both LXRα and LXRβ with or without LPS induction conditions, finding that ILDL exhibited a greater effect on LXRα than LXRβ. Then, we revealed that ILDL significantly reduced the degradation of LXRα by pronase in a dose-dependent manner, confirming its binding ability with LXRα by using DARTS technology. Our study further demonstrated that ILDL significantly increased the expression of LXRα target genes ABCA1 and ABCG1 under normal culture conditions, indicating its ability to activate the LXRα pathway. Additionally, Molecular docking analysis demonstrates a strong binding affinity between ILDL and LXRα protein, with a binding energy of 8.106 kcal/mol, facilitating their interaction. Further analysis and prediction showed that ILDL forms stable bonds with LXRα protein within a cavity hydrophobic amino acids, including phe257, Thr258, Ile295, Met298, Leu299, Thr302, Phe335, and Trp443. This hydrophobic interaction promotes the stability of ILDL-LXRα. These findings collectively indicate that ILDL binds to LXRα and activates LXRα-related pathways.

Numerous studies have highlighted the role of LXRα pathway activation in modulating NF-κB, MAPK pathway, and lipid efflux and metabolism in macrophages, affecting immune responses, inflammation, apoptosis, and phagocytosis [29, 30]. Specifically, LXRα activation inhibits inflammatory genes such as iNOS, IL-6, and IL-1β in macrophages by interfering with NF-κB action on proinflammatory gene promoters. In this study, ILDL significantly reduced LPS-induced NLRP3 expression and PARP split-mediated damage, along with inhibiting ERK phosphorylation. These results suggest that ILDL can directly activate LXRα, exerting LXRα-mediated effects, including inhibiting M1 differentiation and reducing the expression of inflammatory mediators. Subsequent inhibition studies using the LXRα inhibitor GSK2033 further supported the anti-inflammatory mechanism of ILDL, showing a dose-dependent reduction in IL-6 production. Moreover, ILDL significantly inhibited iNOS mRNA expression under normal conditions, with its effect negated when LXRα activity was inhibited by GSK2033. Collectively, these findings indicate that ILDL inhibits macrophage polarization and inflammatory mediators’ production through the LXRα pathway.

Activation of LXRα in macrophages not only mediates anti-inflammatory effects but also promotes macrophage efferocytosis. Firstly, we co-cultured RAW264.7 cells and MODE-K cells by a transwell co-culture system in vitro and found that ILDL could reduce intestinal epithelial cell damage by inhibiting macrophage polarization and inflammatory mediator production. Subsequently, to investigate the effect of ILDL-activated LXRα on macrophage efferocytosis, we simulated the effect of macrophages on apoptotic intestinal epithelial cells in the UC state. We observed that ILDL not only increased the number of macrophages initiating efferocytosis but also enhanced the phagocytosis of apoptotic epithelial cells by macrophages. This demonstrated a significant pharmacological effect of ILDL in promoting efferocytosis. Macrophages then play a pivotal role in phagocytosing apoptotic cells. Efferocytosis can not only effectively remove dead cells to prevent secondary necrosis, avoid tissue inflammation, but also induce metabolic reprogramming of macrophages, inhibit inflammatory M1 polarization, promote anti-inflammatory M2 polarization and tissue repair. Thus, Efferocytosis mediated by LXRα activation has shown promise in improving various inflammatory and immune-mediated diseases such as IBD induced by DSS and TNBS [31]. In the end, we investigated the pharmacological effect of ILDL against UC and found that it effectively improves UC symptoms and tissue lesions, suggesting a good anti-UC effect of ILDL. Furthermore, immunohistochemical and western blot results showed that ILDL decreased the M1 marker CD86 of macrophages and increase M2 marker CD206. Transcriptomic results further confirmed ILDL's significant anti-UC effect, indicating its protective role in regulating the chemotactic infiltration of immune cells such as neutrophils and macrophages during the acute phase. These findings indicate that ILDL effectively inhibits M1 polarization of macrophages both in vivo and in vitro, improves UC disease symptoms and tissue lesions, exhibiting promising anti-UC effects.

The therapeutic landscape for UC features 5-aminosalicylic acid (5-ASA) as a cornerstone treatment for mild-to-moderate cases, while LXR agonists represent an emerging class with distinct mechanistic advantages and challenges [32, 33]. 5-ASA effectively induces remission with localized action and established safety, but its limited immunomodulation fails one-third of patients [34]. In contrast, LXR agonists uniquely combine anti-inflammatory and pro-resolving effects through NF-κB suppression and cholesterol homeostasis regulation, offering potential for refractory cases [31, 35]. However, first-generation LXR agonists face metabolic side effects, driving development of gut-selective variants. Together, these agents represent complementary approaches: 5-ASA for early disease and LXR modulators for complex cases, with future work needed to optimize combination strategies [36].

The current findings are accompanied by limitations. Although phytochemical analysis of *Lindera aggregata* was conducted to identify key components, a comprehensive characterization remains unachieved, highlighting the need for deeper exploration of its chemical constituents [37]. Furthermore, while ILDL exhibits significant anti-inflammatory activity, its bioactive structure and functional groups remain undefined. Given the reported antitumor efficacy of ILDL, future studies should prioritize toxicological evaluations to establish its clinical safety profile [38]. Despite these limitations, our findings demonstrate that ILDL activates the LXRα signaling pathway and ameliorates ulcerative colitis. This work also advances the understanding of *Lindera aggregata's* pharmacodynamic properties, providing valuable pharmacological data to support its translational development.

## Conclusion

In summary, ILDL activates the LXRα pathway, inhibiting macrophage M1 polarization, reducing inflammatory mediator levels, and promoting macrophage efferocytosis. ILDL is a promising candidate compound from *Lindera aggregata* for anti-inflammation and UC treatment.

## Supplementary Information

Supplementary file 1.

## Data Availability

The datasets used and/or analyzed during the current study are available from the corresponding author on reasonable request.

## Abbreviations

UC: Ulcerative colitis
DSS: Dextran sodium sulfate
TNBS: 2,4,6-Trinitrobenzene sulfonic acid
ILDL: Isolinderalactone
LPS: Lipopolysaccharide
NC: Normal control
MC: Model control
SASP: Sulfasalazine
LXR: Liver X receptor
DAI: Disease activity index
PAS: Periodic acid-schiff
H&E: Hematoxylin and eosin
MLNs: Mesenteric lymph nodes
IL-6: Interleukin 6
IL-1β: Interleukin 1β
NO: Nitric oxide
TNF-α: Tumor necrosis factor-α
DARTS: Drug affinity responsible target stability
DEGs: Differential expression genes
PCA: Principal component analysis
ABCA1: ATP binding casstte transporter A1
ABCG1: ATP binding cassette subfamily G member 1
NF-κB: Nuclear factor-κB
MAPK: Mitogen-activated protein kinase
MFI: Mean fluorescence intensity
DAPI: Nuclear dye 4′,6-diamidino-2-phenylindole
DMEM: Dulbecco's Modified Eagle Medium
DMSO: Dimethyl sulfoxide
FBS: Fetal bovine serum
GAPDH: Glyceraldehyde 3-phosphate dehydrogenase
HRP: Horseradish peroxidase
PCR: Polymerase chain reaction
TLR: Toll-like receptor
