# Supplementary material for: Isolinderalactone regulates macrophage polarization and efferocytosis by activating the LXRα pathway against ulcerative colitis
Source: Chin Med. 2025 Oct 1;20:152. doi: 10.1186/s13020-025-01216-9 (PMC12487359; doi:10.1186/s13020-025-01216-9)
Supplement: Supplementary file 1 [file 13020_2025_1216_MOESM1_ESM.docx]

***Supplementary information***

**Isolinderalactone regulates macrophage polarization and efferocytosis by activating LXRα pathway against ulcerative colitis**

**List of Supplementary Materials**

Table S1 to S3

Figure S1 to S2

**Supplementary Table S1. Reagent list used in this study.**

| Reagent | Source | | Catalogue |
| --- | --- | --- | --- |
| Isolinderalactone | Chengdu Desite Bio-Technology | DY0230 | |
| Linderane | Chengdu MUST Bio-Technology | A0367 | |
| Hydroxylinderstrenolide | Chengdu MUST Bio-Technology | A1339 | |
| Lindenenyl acetate | Chengdu MUST Bio-Technology | A1319 | |
| Linderalactone | Chengdu MUST Bio-Technology | A1321 | |
| Lindenenol | Chengdu MUST Bio-Technology | A1320 | |
| Norisoboldine | Chengdu MUST Bio-Technology | A0538 | |
| Dextran sulfate sodium | MP Biomedical | 9011-18-1 | |
| Lipopolysaccharide | Sigma-Aldrich Chemical | 457627 | |
| Sulfasalazine | Sigma-Aldrich Chemical | [PHR3243](https://www.sigmaaldrich.cn/CN/zh/product/sial/phr3243) | |
| CBA Mouse Inflammation detection kit | BD Biosciences | 552364 | |
| phospho-ERK(Thr202/Tyr204) antibody | Cell Signaling Technology | 4370 | |
| ERK antibody | Cell Signaling Technology | 4695 | |
| NLRP3 antibody | Cell Signaling Technology | 15101 | |
| cleaved PARP antibody | Cell Signaling Technology | 9541 | |
| PARP antibody | Cell Signaling Technology | 9542 | |
| α-tubulin antibody | Abcam | ab7291 | |
| GAPDH antibody | Abcam | ab8245 | |
| Occludin antibody | Abcam | ab216317 | |
| β-actin antibody | Abcam | Ab8226 | |
| Annexin V-APC/PI apoptosis detect Kit | Multi Sciences | AP107 | |
| CD206 antibody | HUABIO | ET1702-04 | |
| LXR-α antibody | HUABIO | ET1704-51 | |
| Multicolor protein marker | Vazyme Biotech | MP102-01/02 | |
| Pronase | Roche | [PRON-RO](https://www.sigmaaldrich.cn/CN/zh/product/roche/pronro) | |

**Supplementary Table S2. Primer sequences for qPCR.**

| Gene | Species | Squence |
| --- | --- | --- |
| *Gapdh* | Mouse | TGTGAACGGATTTGGCCGTA  ACTGTGCCGTTGAATTTGCC |
| *Il6* | Mouse | TCCAGTTGCCTTGGGAC  AGTCTCTCCGGACTTGT |
| *Tnfα* | Mouse | GGGCAGGTCTACTTTGGAG  CACTGTCCCAGCATCTTGT |
| *inos* | Mouse | GGAATCTTGGAGCGAGTTG  GAGGGCTTGGCTGAGTGAG |
| *Lxrα* | Mouse | TGTGGAAGACAGAACCTCA  CCAACCCTATCCCTAAAGC |
| *Lxrβ* | Mouse | CGCTACAACCACGAGACAG  GGCGATAAGCAAGGCATAC |
| *Abca1* | Mouse | CAGAAAACCGCAGACATCC  GAAAACCCGCCATACCGAA |
| *Abcg1* | Mouse | ACTGCCCTACCTACCACAA  CAAAGAAACGGGTTCACAT |

**Supplementary Table S3. Specific siRNA sequences.**

| Gene | Species | Squence |
| --- | --- | --- |
| *Lxrα* | mouse | GUGUCAUCAAGGGAGCACGCUAUGUTTT  ACAUAGCGUGCUCCCUUGAUGACACTT |

**Supplementary Figures and Figure Legends**


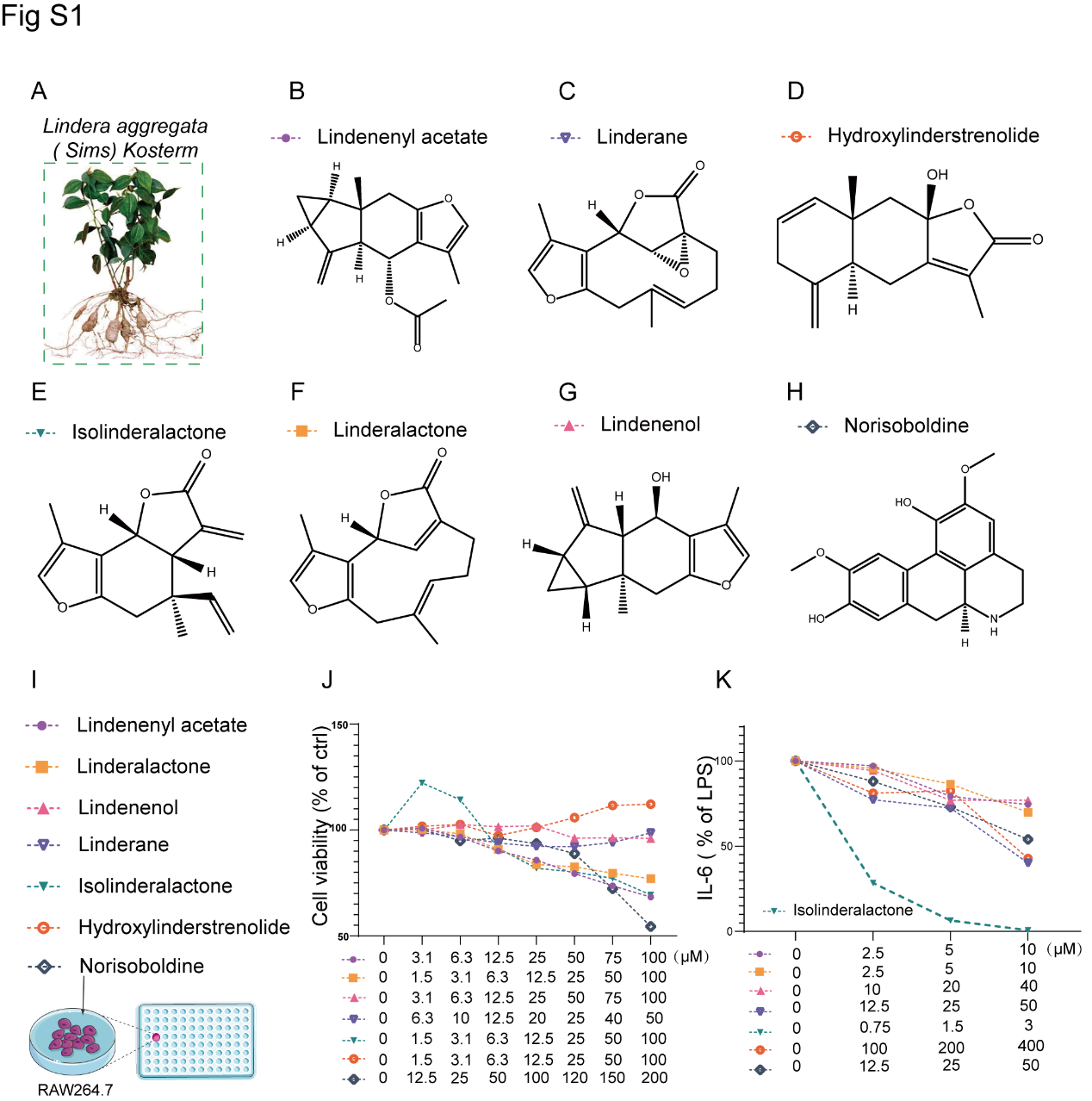


**Supplementary Fig. S1. Isolinderalactone (ILDL) exhibits excellent anti-inflammatory activity among many known characteristic components of *Lindera aggregate (Sims) Kosterm.***

**A** *Lindera aggregate (Sims) Kosterm.* **B-H** The chemical structure of Lindenenyl acetate， Linderane， Hydroxylinderstrenolide, Isolinderalactone, Linderalactone, Lindenenol and Norisoboldine. **I** Schematic diagram of the screening design for compounds using the RAW264.7 cells. **J** The RAW264.7 cells viability after 48h intervened by the above compounds respectively. **K** The relative percentage of IL-6 in LPS-stimulated RAW264.7 cell supernatants after 48h intervened by the above compounds respectively (n = 3).

**Supplementary Fig. S2.**


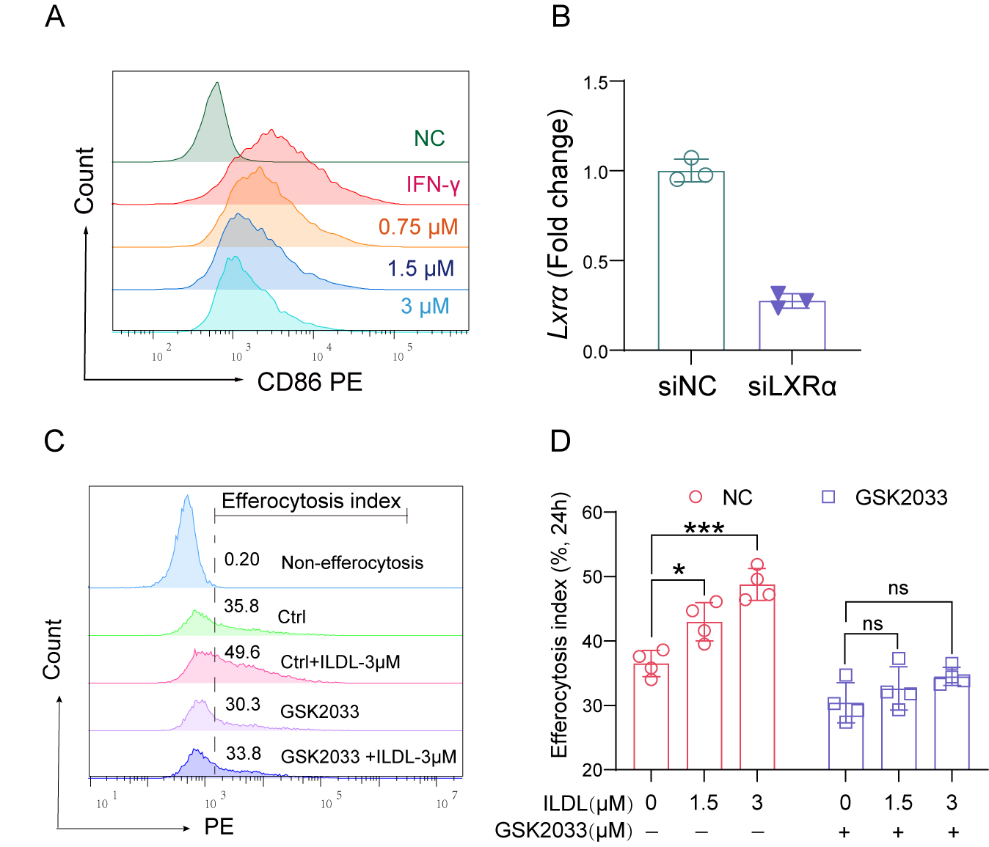


**Supplementary Fig. S2.**

**A** Histogram of macrophage M1 polarization induced by IFN-γ. **B** Verification of siLXRα silencing efficiency (n = 3). **C-D** Effect of ILDL on efferocytosis under normal and LXRα-silenced conditions (n = 4).
